# Acute Epiphysitis in Infants—Nontubercular1Read by invitation before the Medical Society of the County of Erie, June 9, 1895.

**Published:** 1896-10

**Authors:** Nelson G. Richmond

**Affiliations:** Fredonia, N.Y.


### BUFFALO MEDICAL JOURNAL.

VOL. XXXVI. OCTOBER, 1896. No. 3.

### Original Communications.

### ACUTE EPIPHYSITIS IN INFANTS—NONTUBERCULAR.[1]

By NELSON G. RICHMOND, M. D., Fredonia, N.Y.

ACUTE epiphysitis is an acute inflammation of the epiphyseal ends of bones before they are united to the shaft or diaphysis. It is a very rare disease. It is very fatal.

On February 7, 1896, I was called to see Edith J., aged 5 months, who, with her twin sister Marjorie, was suffering from acute indigestion and diarrhea. A change of diet was followed by immediate improvement. February 11th I was called again to see Edith and found her with some fever and a swollen right knee, which was painful on motion. A diagnosis of rheumatism was made and she was placed upon five-grain doses of sodium salicylate. On February 12th and 13th she showed improvement. The case was not under observation for the next week.

February 21st not feeling at rest in my mind, in the absence of any report, I called at the house as a precaution. I found that an abscess had broken that morning, the sinus opening just above the patella. There was also fluctuation just below the head of the tibia. A free incision was made over the point of fluctuation and an ounce or two of pus obtained. The bottom of the abscess was so near the posterior surface that a counter opening was made in the calf of the leg. Thus drainage was obtained directly through the leg. Both places were syringed with hydrogen peroxide ; iodoform gauze was used for drainage and constitutional remedies and diet were prescribed. There was an apparent physical improvement and closing in of the sinuses—the posterior one being allowed to heal—until March 7th, when the knee was found more swollen and darker in color. The sinuses were syringed with a solution of permanganate of potash. March 8th, improvement. March 9th, called 4 A. M., baby for a few hours had been growing restless and was crying. The leg was swollen and the left iliac region was hard. On dressing the wounds drainage was found deficient in the upper sinus. In the evening consultation was held with Dr. A. W.

1. Read by invitation before the Medical Society of the County of Erie, June 9, 1896.

Dods. The abdomen was then found tympanitic over the entire region and a marked loss of strength was apparent from the morning visit. Free incisions with a view of establishing freer drainage were decided upon as the only measure which might avail in saving the child's life.

March 10th, found little patient with a weak pulse of 180 and respiration of 80. Veins were blue and full. There was projectile vomiting; pallor marked and symptoms of final dissolution were prominent. With Dr. Moore assisting us and a perfect understanding that the child might not survive the anesthetic or operation and probably would not live twenty-four hours, chloroform was given. Under it the pulse came down to 120 with no loss of strength. An incision was made in each case down to the tibia and femur; the periosteum was split and the incision carried to the medullary canal. Evening, no loss of strength from the operation. Condition no worse. March 11th, pulse, 175 to 200 and weak; respiration, 60 to 90. Sleeping quietly most of the time. Taking nourishment. Wound dry. Hot bichloride dressing used and changed frequently. March 12th, pulse, 160 to 180; respiration, 50 to 80. Syringed with hydrogen peroxide. Leg still swollen and cellular tissue involved. Secretion from wounds scanty. A voluntary movement from bowel. March 13th, pulse, 160 to 186; respiration, 50 to 72. A zone of hardness and redness extending over descending colon has been apparent for past forty-eight hours and is now marked. The cellulitis of the leg has increased. Incisions are not secreting as well as hoped. The general peritonitis is apparently giving place to the circumscribed cellulitis. The incisions were washed daily with peroxide and hot bichloride dressing kept up. Hot dressings of cotton, saturated with hamamelis were kept over the region of the cellulitis. At 4 P. M., has vomited the barley gruel and top-milk mixture, also the egg albumin. Quieter than yesterday.

March 14th, A. M., pulse, 150 to 176; respiration, 50 to 60; a quiet night; no vomiting; omitted dressing; less cellulitis in leg; abdomen still hard and red; a voluntary passage; witch-hazel dressing kept up. Night, pulse, 150; respiration, 45; moderate vomiting; child quiet.

March 15th, A. M., a good night; pulse, 130 to 150; respiration, 40 to 55; a reduction in frequency of both pulse and respiration from day to day. Coughing causes pain, although baby is moved with less pain. The cellulitis is disappearing from abdomen. The leg is less swollen and the wounds are healthy; no pus; dressing omitted. Night, pulse, 160 to 184. Vomited once today. Pulse highest at time of vomiting.

March 16th, washed wounds with peroxide and dressed with hot bichloride—the first time in three days. No pus. Wounds covered with healthy granulations. Improvement in swelling of the leg and groin. Night, pulse, 144 to 156; respiration, 40 to 50; a good day.

March 17th, was called early to see little patient. She had vomited twice during the night and was very drowsy. Had slept eighteen hours out of the twenty-four. There was extreme pallor, and yet a dark hue to skin of the face. At times breathing quietly and 40 a minute. Next minute catchy and 90 to 100. Pulse, 150; then 180, pounding and remittent. Opened wounds and found lower one clean and upper one producing pus. I incised again to the bone, washed with peroxide and dressed. 10 A. M. there was a convulsion. Fontanelles distended. Still in stupor. Respiration one minute, 148 to 160, next minute 50. Noon, failing. Stupor more marked. One more convulsion. Died at 1.30 P. M., apparently from septic meningitis.

In looking up the literature of the subject I have considered acute "arthritis of infants," "acute osteitis of infants," "acute osteomyelitis," "acute epiphysitis of infants" and "periostitis."

In Volume XXXI. of the *Medical Record*, 1887, Dr. V. P. Gibney reports a case beginning at six months of age and dying at four years and three months of tubercular meningitis. An autopsy confirmed the diagnosis. A few scattered tubercles were found in each lung. It is more than likely that this was tubercular from the beginning. If so, it hardly belongs to the class of cases being described. Gibney follows European observers in using the term epiphysitis. He also quotes Thomas Smith, who in 1874 reported ten cases in which the hip-joint was affected.

The Cyclopedia of the Diseases of Children, edited by Keating, published in 1890, gives a list of thirty-four cases. Undoubtedly the cases above mentioned are included in the list of Keating's. The mortality of these thirty-four cases was 45 per cent. One of the infants required an amputation of the leg. The ages were four, eight, ten, twelve and nineteen days to thirteen months.

Under Periostitis, Ashby and Wright, edition of 1896, describe a class of cases rare in infancy and not occurring in adult life. They mention it as having been seen only twice under two years old.

Watson Cheyne mentions a case of Rosenbach's in which periostitis occurred in utero, but this was considered to be an acute osteomyelitis "epiphysitis."

Mr. McNamara, Mr. Tubby and others believe that all these cases really begin as an inflammation of the epiphyseal line and that the mischief spreads downward and upward both beneath the periosteum and in the medulla. Ashby and Wright deny that this is the universal way, although allowing that it does occur.

Ashby and Wright also describe a syphilitic epiphysitis as occurring near the epiphyses in the long bones, especially at the lower ends of the humerus, femur, radius and tibia, in infants who are suffering severely from hereditary syphilis. They also differ from all the authors consulted, who either say nothing or who speak of its rarity, by saying that acute osteomyelitis or acute epiphysitis is not an uncommon condition.

Under acute suppurative arthritis of infants, they give credit to T. Smith, of St. Bartholomew's, for first describing the disease, and speak of it as of fairly frequent occurrence.

I desired to get some idea of the frequency of this disease in private and hospital practice at the present time, at least the frequency of its recognition ; also the proportion of cases of acute epiphysitis to births. Accordingly I addressed twenty-five letters to some of the leading physicians in general and obstetrical practice in Buffalo, Syracuse, New York and towns throughout the state. I also made a somewhat similar inquiry of some specialists in pediatrics. I received in reply definite answers from physicians having had from 100 to over 3,000 cases of obstetrics in their private and hospital work.

The total number of obstetrical cases thus reported was over 14,000. Some with very large obstetrical practices and holding the chair of obstetrics in the first colleges of the state, report having had no cases of epiphysitis. Not having given their obstetrical experience in figures the above summary does not include their list. No one of those reporting the above 14,000 births could recall a case of epiphysitis, or was aware that they ever had one, except Dr. J. G. Thompson, of Angola, N. Y. He reported two cases : one, an Indian child of two months, which was lost sight of ; another, a white child, was born with the disease and died at three months of phthisis.

The service of Dr. Lake, of Gowanda, N. Y., in the Cattaraugus Indian reservation, leads him to say that tuberculosis of today is only a sequel to syphilis a decade ago. From the lack of continued observation and the history, it is more than probable that this Indian child had the tubercular form ; or it might have had epiphysitis from hereditary syphilis. The white child was born with the disease and died at three months of phthisis. In case they both are tubercular they should be excluded from this list.

Dr. Louis Fischer, whose specialty is pediatrics, seems to have an experience differing from the other observers, as he reports the number of cases in his hospital and dispensary practice as being 10 per cent. He quotes ages, however, up to 9 and 10 years. He thus confirms the quotations already made from Ashby and Wright as to frequency. Fischer believes epiphysitis to be connected with or found with "growing pains" of children and usually relieved by salicylic acid. In this opinion he is backed by no less an authority than Ashby and Wright, who describe "growing fever" under the head of epiphysitis as found in children from 7 to 15 years.

From the fact that this paper is dealing with the disease in infants and that infancy can hardly be said to extend beyond two years and that Ashby and Wright in all their classification describe the disease without designating infancy, the clashing of these authorities with some of our conclusions is not as great as would appear.

Of the thirty-four cases reported in Keating's Cyclopedia the hip was primarily affected in 11 cases, the knee in 14, the shoulder in 5. In one case both hip-joints were involved when first seen; in one the hip and knee, and in an infant twelve days old many of the epiphyses were found infiltrated with pus in post mortem.

The disease most commonly attacks the head of the femur, the upper end of the tibia or the lower end of the femur. The cause has been described in only two or three instances. In these it was traumatic. The disease begins as an acute congestion of the blood-vessels of the epiphysis, quickly resulting in inflammation and pus. The pus may quickly involve the joint, even by a pin-hole opening and is the more likely from its proximity, the cartilaginous structure of the epiphysis and the fact that the epiphysis, in infancy, is incorporated in the synovial sheath.

The pus without invading the joint, or even after it has occurred, may burrow along under the periosteum, breaking through at some distant point. Or it may involve the diaphysis, or some neighboring joint. Or it may be absorbed into the general system, as in the case related, resulting in peritonitis and meningitis. The staphylococcus aureus and albus have been demonstrated in bones affected with this disease and it is probably due to these microbes that the general invasion and sepsis occurs. Necrosis of the epiphysis quickly follow this destructive change. If the child survives the attack, deformities and subluxation usually result; rarely anchylosis, in those cases where the joint has been involved.

The epiphysis has been explained as the starting point of the disease, as it is here that the centers of ossification are the most vascular. Thus far investigation seems to show that the pathology is the same as that of acute osteomyelitis. Others say acute infectious osteomyelitis. Others still, that it is a pyemia of bone. The disease runs a rapid course. Cases have proved fatal in a week. Often an abscess forms and opens within this time. The symptoms ushering in the disease are fever and restlessness, pain on being moved and swelling of the joint. There is flexion of the affected limb. The disease with which it is most likely to be confused is acute articular rheumatism. At first it may be impossible to decide. Epiphysitis is more apt to involve one joint; is less migratory ; is more rapid in its course and in case of suppuration the skin could be expected to assume a darker color. The differentiation will probably have been made before pyemic complications arise.

One aim of this paper is to come to the conclusion whether there is an epiphysitis nontubercular or not. This case cited was a well-nourished child, one of twins and the stronger one, but there was no history of tuberculosis on either the father's or mother's side. From the well-established fact of the hereditary nature of the constitution which readily gives fertile ground for the bacillus tuberculosis and from the cases with a tubercular history, it is very certain that there is an epiphysitis of a tubercular nature and it is probably more common than the nontubercular form.

Fischer considers many cases tubercular, others rachitic and finds the disease mostly in bottle-fed babies. Gibney says : "There is only one case reported where it has been found that the disease was tubercular." A. B. Judson says : "It may therefore be believed that some of the cases of so-called diastasis attended with suppuration are simply instances of an acute epiphysitis. Keating's work describes this disease under "Nontubercular disease of joints." It undoubtedly voices the truth when it says : "There is no question that there is an epiphysitis of tubercular origin, in fact many cases of strumous disease of the bones entering into the formation of joints begin in or about the epiphysitis, but the class of cases now referred to do not belong to this category." Ashby and Wright describe the disease fully, but do not distinguish between the disease of infancy and childhood, nor between the tubercular and nontubercular form. Hirsch does not mention epiphysitis in the edition which I have. Osler's Practice of Medicine, under the title, Acute necrosis, or Acute osteomyelitis, speaks of the condition as sometimes occurring in the lower end of the femur or head of the tibia and as being sometimes mistaken for rheumatism. One observer gives under the head of causes or varieties : (1) Tubercular, including the so-called strumous diathesis; (2) Syphilitic ; (3) Injury during delivery by traction or subsequent thereto.

The successful treatment of this disease depends upon an early diagnosis. The constitutional treatment is such as would present itself to any intelligent practitioner. Jacobi, theoretically, would advocate phosphorus internally, if cases of a subacute variety could be found.

The real treatment is surgical. A free incision should be made down to the bone, the periosteum slit and the bone or cartilage freely opened. Free drainage is the thing to be attained ; hot bichloride dressings are the best to maintain it. This incision should be made early. A delay of twenty-four hours may mean the involvement of a joint. The nidus may be in the periosteum, or underneath the epiphyseal substance or in the diaphyseal side, but the nearness to the joint, the epiphysis being included in the synovial sheath and the cartilaginous character of it, make it absolutely essential to establish free drainage if the joint is to be saved.

The necessity for early action appeared in the case cited. The opening above the knee apparently gave rise to all the subsequent trouble. The incision below the knee, which was made early, was very tractable. From the authorities cited, from the expression of opinion of those sought; from the rapidity of the inflammation extending to and involving the joint ; from the fact that it presents all the symptoms of acute osteomyelitis, resulting in early necrosis, I believe that we are justified in maintaining that (1) There is an acute epiphysitis in infants of a nontubercular form ; (2) That there is a tubercular, also a syphilitic form, both hereditary, in infancy and childhood ; (3) That epiphysitis is a rare disease in infancy and a more common disease in childhood ; (4) That both the tubercular and nontubercular forms are more common in hospital than in private practice ; (5) That undoubtedly many cases of arthritis are primary epiphysitis.

Since writing this paper I have received a reprint from the pen of my friend Dr. Wisner R. Townsend, of New York, the original article being published in the *American Journal of the Medical Sciences* of January, 1890. The paper is on Acute arthritis of infants. He prefers this title, although he describes the epiphysis as the starting point and quotes many of the authorities this paper has. From his own experience in the Hospital for the Ruptured and Crippled during the previous year and the experience of his friends, he gives the history of nineteen cases. Four of these nineteen cases give clear histories of phthisis in one or both parents, or a near relative. In others the history on this point is negative. Six cases were fatal and in two more the results were not given. He reports in all seventy-one cases including his own. This number includes the greater portion of those reported in this paper. The mortality of the seventy-one cases is forty-five per cent. His paper is an admirable one and his conclusions do not clash with the conclusions of this paper. It is only a matter of surprise that he has the opportunities for studying so large a number of cases, in so short a period, when the disease is so uncommon and the literature so scanty and uncertain.

